# Identification and Validation of HCC-specific Gene Transcriptional Signature for Tumor Antigen Discovery

**DOI:** 10.1038/srep29258

**Published:** 2016-07-08

**Authors:** Annacarmen Petrizzo, Francesca Pia Caruso, Maria Tagliamonte, Maria Lina Tornesello, Michele Ceccarelli, Valerio Costa, Marianna Aprile, Roberta Esposito, Gennaro Ciliberto, Franco M. Buonaguro, Luigi Buonaguro

**Affiliations:** 1 Laboratory of Molecular Biology and Viral Oncology, Naples-Italy; 2 University del Sannio, Benevento-Italy; 3 BIOGEM S.c.a.r.l., Ariano Iprino-Italy; 4 Institute of Genetics and Biophysics “A. Buzzati-Traverso” (IGB), National Research Council, 80131 Naples, Italy; 5 Scientific Direction, Istituto Nazionale per lo Studio e la Cura dei Tumori, “Fondazione Pascale”-IRCCS, Naples-Italy

## Abstract

A novel two-step bioinformatics strategy was applied for identification of signatures with therapeutic implications in hepatitis-associated HCC. Transcriptional profiles from HBV- and HCV-associated HCC samples were compared with non-tumor liver controls. Resulting HCC modulated genes were subsequently compared with different non-tumor tissue samples. Two related signatures were identified, namely “HCC-associated” and “HCC-specific”. Expression data were validated by RNA-Seq analysis carried out on unrelated HCC samples and protein expression was confirmed according to The Human Protein Atlas" (http://proteinatlas.org/), a public repository of immunohistochemistry data. Among all, aldo-keto reductase family 1 member B10, and IGF2 mRNA-binding protein 3 were found strictly HCC-specific with no expression in 18/20 normal tissues. Target peptides for vaccine design were predicted for both proteins associated with the most prevalent HLA-class I and II alleles. The described novel strategy showed to be feasible for identification of HCC-specific proteins as highly potential target for HCC immunotherapy.

Hepatocellular carcinoma (HCC) is the third leading cause of cancer-related death worldwide with a 5-year survival rate of approximately 5–6%^1^,^2^. Multiple etiological factors for HCC are known with an uneven geographical distribution. Globally, more than 50% of HCC cases can be attributed to hepatitis B virus (HBV) infection, more than 30% to hepatitis C virus (HCV) infection and approximately 15% can be associated with non-viral causes (i.e., alcohol, aflatoxins, metabolic liver diseases, steatosis and non-alcoholic fatty liver disease)^3^.

Regardless the etiology, the overall prognosis for HCC patients is poor with a median survival of 14 months and less than 5% of symptomatic patients surviving more than 2 years^1^,^2^. A range of therapies are used in the management of HCC according to the extent and severity of liver disease. In early-stage HCC, surgery (i.e., tumor resection and liver transplantation) represents the standard treatment with a 5-year survival rate in 70% of treated patients^4^,^5^,^6^. However, the majority of HCC patients are diagnosed when disease precludes surgical approaches and only loco-regional therapies can be applied with limited survival benefits. Moreover, tumor recurrence is observed in 50 to 80% of patients at 5 years after treatment, with most relapses occurring within 2 years^7^. Therapeutic options in advanced unresectable HCC are limited to Sorafenib which is the only approved therapy confirmed to provide a limited increase in survival of 2.3–2.8 months^8^,^9^,^10^,^11^.

In this framework, immunotherapeutic interventions, including cancer vaccines, may represent a novel and effective therapeutic tool for HCC. However, only few clinical trials have been conducted so far, showing efficient elicitation of immune response with limited clinical outcomes. One of the major reasons for such unsatisfactory results is due to low number of tumor antigens and epitopes specific for HCC to be employed in active and passive immunotherapy approaches^12^. Indeed, identification of “tumor-specific” target molecules suitable for therapeutic strategies represents a primary goal and a major challenge.

In this perspective, DNA microarrays represent a powerful tool. However, all published studies are designed to compare transcriptional profiles of tumor and normal counterpart of the same organ/tissue, aiming at identifying genes and/or pathways involved in the tumor pathogenesis and progression^13^,^14^. Given that no information is provided concerning their expression levels in other normal tissue types, products of such genes could only be of diagnostic and/or prognostic value for that specific tumor.

On the contrary, in the present study the analysis was designed to identify genes specifically over-expressed in HCC tumor lesions as compared to several non-tumor tissue types. This would lead to discovery of potential HCC-specific targets for immunotherapy with minimal residual chance of toxicity. To this aim, a two-step analysis was conducted. In the first-step, a meta-analysis was performed comparing large datasets of microarray-based transcriptional profiles from virus-associated liver cancer with non-tumor liver controls. The HCC modulated gene set was subsequently compared with transcriptional profiles from several non-tumor tissue types, including liver.

Indeed, three different gene signatures were recognized. The “liver-specific” signature, including genes upregulated in both HCC and normal liver samples as compared to all other non-tumor tissue types. The “HCC-associated” pathogenetic signature, including genes upregulated in HCC tumor lesions as compared to non-tumor liver samples and the majority of non-tumor tissue types. The “HCC-specific” signature, a subset of the “HCC-associated” signature that included genes uniquely upregulated in HCC tumor lesions as compared to all non-tumor liver samples and non-tumor tissue types. Genes included in the “HCC-specific” signature were fully validated by RNA-Seq analysis on unrelated HCC samples and their protein expression was confirmed by interrogating the “The Human Protein Atlas”. Among all, aldo-keto reductase family 1 member B10 (AKR1B10), and IGF2 mRNA-binding protein 3 (IGF2BP3) were strictly HCC-specific with no expression in 18/20 normal tissues. Potential target peptides for vaccine design were predicted for both proteins associated with the most prevalent HLA-class I and II alleles.

## Results

### Identification of “tumor-associated” and “liver-specific” genes

**Figure 1:**
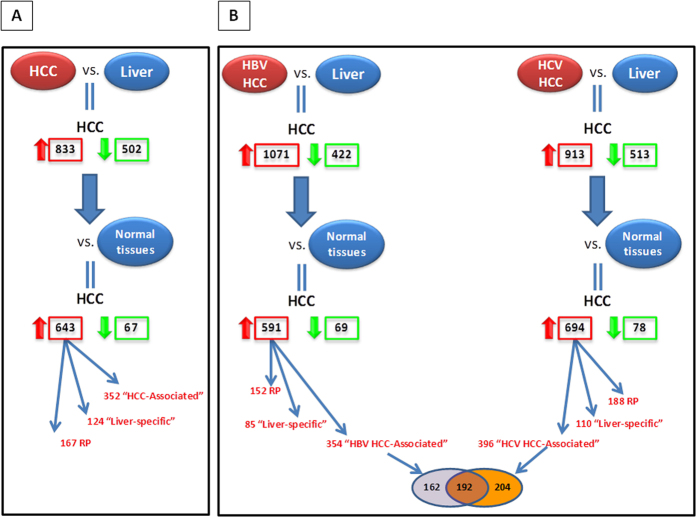
Workflow of the analysis for the identification of the tumor gene transcriptional signature. Identification of “tumor-associated” and “liver-specific” upregulated genes, in HCC (**A**) and in HBV- and HCV-related HCC (**B**). Venn Diagram of “tumor-associated” upregulated genes identified in HBV- and HCV-related HCC (**B**). RP = redundant probes.

**Figure 2:**
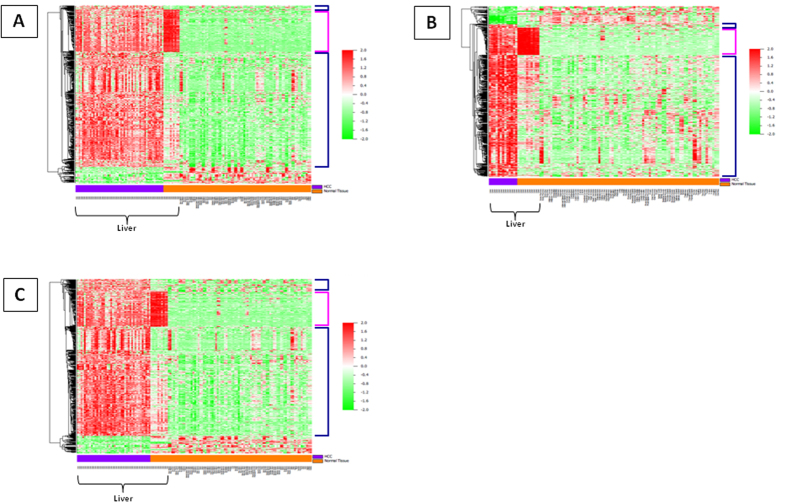
Heat-maps of differentially expressed genes identified in HCC (**A**), HBV-related HCC (**B**) and HCV-related HCC (**C**) vs. normal tissues. Blue bracket = “tumor-associated” upregulated genes. Pink bracket = “liver-specific” upregulated genes. Enlarged list of normal tissues is available at Suppl. Figure 8.

Transcriptional data from HCC (55 samples) and normal control liver (14 samples) were selected from publicly available database and compared for identifying HCC-associated transcriptional patterns. Overall, 1385 genes were differentially expressed. Among them, 883 were upregulated and 502 were downregulated in HCC. Genes differentially modulated were used for subsequent comparison including a broad spectrum of samples from 41 different normal tissue types. Such a second-step analysis identified 710 differentially expressed genes (643 upregulated and 67 downregulated genes in HCC) (Figs 1A and 2A).

The 643 upregulated genes were classified in two distinct signatures: “HCC-associated”, including 352 unique genes specifically upregulated in HCC samples vs. the majority of normal tissue types; and “liver-specific”, including 124 unique genes upregulated in both HCC and normal liver samples (Fig. 1A; Suppl. Table 1).

The same workflow was applied by stratifying the HCC samples according to viral etiology, to verify whether such signatures were common or specific to HBV and HCV pathogenesis (Fig. 1B).

A first comparison analysis between liver tissues from HBV-HCC and normal control liver (i.e., 13 HBV-HCC vs. 14 normal control liver) identified overall 1493 differentially expressed genes. Among them, 1071 genes were upregulated and 422 were downregulated in HBV-HCC. The modulated genes were subsequently used for a second-step comparison with the samples from 41 different normal tissue types. The latter analysis showed that 660 genes were differentially expressed in HBV-HCC samples as compared to normal tissue types (Fig. 2B). Among them, 591 were upregulated and 69 downregulated in tumor samples (Fig. 1B). Within the 591 upregulated genes, the “HBV-HCC-associated” signature included 354 unique genes and the “liver-specific” signature included 85 unique genes (Fig. 1B; Suppl. Table 1).

Similarly, liver tissues from HCV-HCC were compared with normal control liver (i.e., 42 liver tissues from HCV-HCC vs. 14 normal control liver). The first step identified overall 1426 genes differentially expressed. Among them, 913 genes were upregulated and 513 were downregulated in HCV-HCC. The modulated gene set was subsequently used for the second-step comparison with samples from 41 different normal tissue types. Such analysis showed that 772 genes were differentially expressed in HCV-HCC samples as compared to normal tissue types (Fig. 2C). Overall, 694 genes were upregulated and 78 were downregulated in tumor samples (Fig. 1B). Within the 694 upregulated genes, the “HCV-HCC-associated” signature included 396 unique genes and the “liver-specific” signature included 110 unique genes (Fig. 1B; Suppl. Table 1).

For each setting described above, the sum of genes included in the “tumor-associated” and “liver-specific” signatures resulted to be lower than the total number of upregulated genes because of the redundancy of probes related to a single gene on the microarray chips.

Overall, considering the “tumor-associated” signatures stratified for the viral etiology, we identified 192 upregulated genes common to HBV- and HCV-HCC samples, 162 upregulated genes in HBV-HCC and 204 upregulated genes in HCV-HCC (Fig. 1B).

### Analysis of “liver-specific” genes

“Liver-specific” genes identified in the three settings were manually extracted and subjected to gene ontology (GO) enrichment analysis (http://www.geneontology.org). The functional analysis strongly indicated that gene sets characterized by the highest statistical significance converged upon several liver metabolic pathways, including: lipid metabolism, oxidation-reduction process, complement activation (Suppl. Table 2).

IPA analysis showed that the selected upregulated genes were associated with very few activated molecular pathways. In particular, the LXR/RXR Activation pathway was common to all three settings (data not shown).

### IPA analysis of “tumor-associated” genes

#### i. Canonical pathway analysis

Genes included in the “tumor-associated” signatures (i.e., HCC, HBV-HCC and HCV-HCC) were used for IPA analysis to identify common or unique molecular pathways.

Top scoring pathways in the “HCC-associated” signature were the Estrogen-mediated S-phase Entry and Mitotic Roles of Polo-Like Kinase, characterized by a positive z-score of 2, predictive of an overall increase in the activity of the two pathways as compared to normal tissue samples (Suppl. Figure 1).

In parallel, the Estrogen-mediated S-phase Entry pathway resulted as the top scoring also in the “HBV-HCC-associated” signature with a z-score of 1, Cyclins and Cell Cycle Regulation pathway resulted as second top scoring with a z-score of 0.707 (Suppl. Figure 2).

On the contrary, the top scoring pathway in “HCV-HCC-associated” signature was the Interferon Signaling with a positive z-score of 2.646 (Suppl. Figure 3). Additional activated pathways were the p53 Signaling and, common to the previous settings, the Estrogen-mediated S-phase Entry, with a z-score of 2 (Suppl. Figures 1–3).

In addition, the Cell Cycle: G2/M DNA Damage Checkpoint Regulation pathway was characterized by a negative z-score in all three settings, predictive of an overall decrease in its activity in HCC samples, regardless of the viral etiology, as compared to normal tissue samples (Suppl. Figures 1–3).

#### ii. Toxic and molecular/cellular function analysis

A correlation analysis between genes differentially expressed and toxic functions was also performed. IPA showed in all three analysis settings a significant association between modulated gene sets and development of hepatotoxicity and liver disorders, such as Liver Hyperplasia/Hyperproliferation (p-value = 4.82E-03) and Hepatocellular Carcinoma (p-value = 9.44E-04). Such association was further supported by an activation z-score > 2. A significant correlation was observed also with renal toxicity (p-value = 6.15E-03), although with an activation z-score < 2 (data not shown).

In addition, a correlation analysis between differentially expressed genes and diseases and molecular/cellular functions identified in all three analysis settings a significant association with Cellular Growth and Proliferation, and Infectious Diseases, supported by the highest positive z-scores (data not shown).

#### iii. Upstream transcriptional regulators analysis

An upstream transcriptional regulators analysis was performed, in which the activated/inhibited functional state was inferred from the modulation state of several downstream target molecules.

In particular, according to HCC modulated genes, a specific activation of several factors was deduced, including E2F1, ERBB2, CCND1, RABL6, HGF, MYC, VEGF, IRF1, TNF, STAT1, JUN, TRAF2, AKT, WNT1 and CTNNB1. They all play a central role in cell growth, proliferation and survival. Among all, only STAT1 was transcriptionally upregulated (Suppl. Table 3A). Furthermore, the analysis identified several tumor suppressor genes (i.e., CDKN2A, RB1, let-7, RB, KDM5B, BNIP3L, RBL2, NUPR1, STK11) with negative activation z-score, predictive of an inhibited state associated with HCC. Interestingly, in contrast to the predicted inhibition of CDKN2A, the gene was upregulated suggesting a possible loss of function due to inactivating mutations (Suppl. Table 3B).

Interestingly, the tumor suppressor TP53 and the 26 S Proteasome complex were predicted as inhibited only in HBV-related HCC (Suppl. Table 4A,B). Moreover, several members of the IRF family of transcription factors including IRF3, IRF5 and IRF7, together with TLR3 and TLR7 were activated only in HCV-related HCC (Suppl. Table 5A,B)

In addition, the analysis of upstream regulators identified a predicted inhibited state for several microRNAs (miRs). In particular, a specific inhibition of miR-16, miR-146 and the let-7 family of miRs was predicted in HCC lesions. miR-26, miR-124 and miR-155 were inhibited in HBV-associated HCC. miR-24, miR-29, miR-124 and miR-291 were inhibited in HCV-associated HCC (data not shown).

### Identification of “tumor-specific” genes

A manual curation of the “tumor-associated” genes was performed in order to identify a “tumor-specific” signature. In particular, genes were selected as upregulated in >80% of tumor samples with a log_2_ fold change >1 and downregulated or upregulated in <10% of all normal tissue types with a log_2_ fold change <−1.

According to such strategy, “HCC-specific”, “HBV-HCC-specific” and “HCV-HCC-specific” signatures were identified.

**Figure 3:**
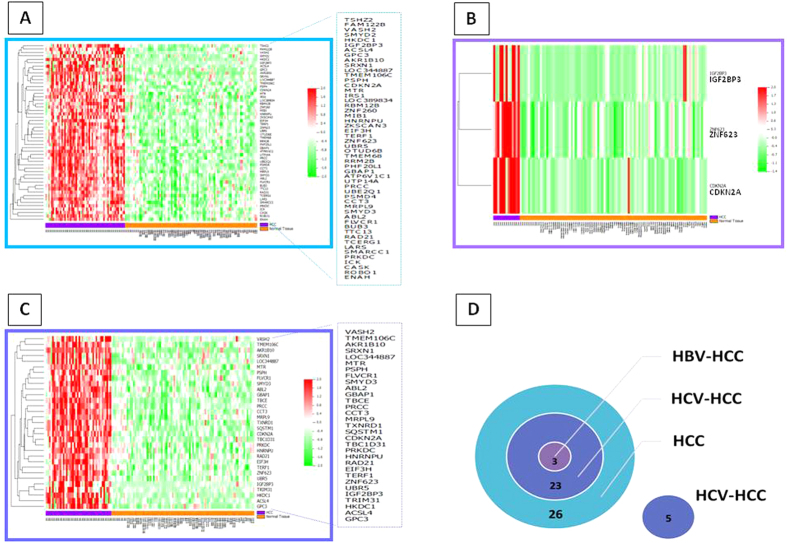
Heat-maps of “tumor-specific” genes identified in HCC (**A**), HBV-related HCC (**B**) and HCV-related HCC (**C**). Number of “tumor-specific” genes that are common or unique to the three analyses (**D**).

The “HCC-specific” signature included the following genes: ABL2, ACSL4, AKR1B10, ATP6V1C1, BUB3, CASK, CCT3, CDKN2A, EIF3H, ENAH, FAM122B, FLVCR1, GBAP1, GPC3, HKDC1, HNRNPU, ICK, IGF2BP3, IRS1, LARS, LOC344887, LOC389834, MIB1, MRPL9, MTR, OTUD6B, PHF20L1, PRCC, PRKDC, PSMD4, PSPH, RAD21, RBM12B, ROBO1, RRM2B, SMARCC1, SMYD2, SMYD3, SRXN1, TCERG1, TERF1, TMEM106C, TMEM68, TSHZ2, TTC13, UBE2Q1, UBR5, UTP14A, VASH2, ZKSCAN3, ZNF260, ZNF623 (Fig. 3A). Whereas, the “HBV-HCC-specific” signature included three genes, namely CDKN2A, IGF2BP3 and ZNF623 (Fig. 3B). Finally, the “HCV-HCC-specific” signature included: ABL2, ACSL4, AKR1B10, CCT3, CDKN2A, EIF3H, FLVCR1, GBAP1, GPC3, HKDC1, HNRNPU, IGF2BP3, LOC344887, MRPL9, MTR, PRCC, PRKDC, PSPH, RAD21, SMYD3, SQSTM1, SRXN1, TBC1D31, TBCE, TERF1, TMEM106C, TRIM31, TXNRD1, UBR5, VASH2, ZNF623 (Fig. 3C).

Interestingly, the three “HBV-HCC-specific” genes were common to the HCV-HCC, suggesting the identification of potential molecular targets irrespective of the viral etiology (Fig. 3D).

The network analysis showed that most of the “tumor-specific” genes identified in the three settings were related to cancer, cell cycle, cell morphology and movement, DNA replication and inflammatory response. In particular, the CDKN2A gene was associated with the “Cell Cycle, DNA Replication, Recombination and Repair, Cellular Response to Therapeutics” network, in the HBV-HCC setting (Suppl. Figure 4), and the “Cell Cycle, Post-Translational Modification, Cellular Development” network in the HCV-HCC setting (Suppl. Figure 5). Interestingly, the same networks also included IGF2BP3 (Suppl. Figures 4 and 5). On the contrary, the ZNF623 gene was associated with the “Developmental Disorder, Hereditary Disorder, Organismal Functions” network, in the HBV-HCC setting, and the “Antimicrobial Response, Inflammatory Response, Dermatological Diseases and Conditions” network, in the HCV-HCC setting (Suppl. Figures 6 and 7). The genes included in these networks are listed in supplementary Table 6.

**Figure 4:**
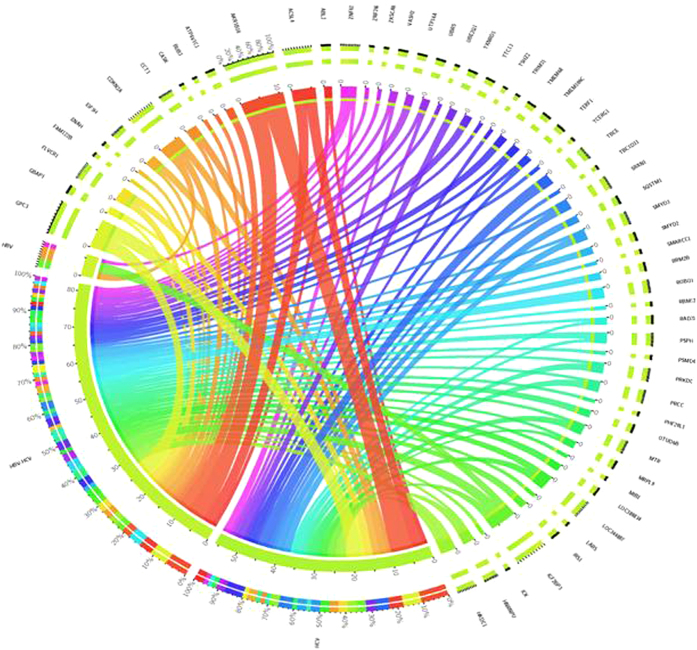
Circos plot showing log_2_ fold change of “tumor-specific” genes. Log_2_ fold changes for each of the genes identified in the “tumor-specific” settings/signatures are indicated by the width of ribbons. Presence of each gene in the “tumor-specific” settings/signatures is indicated by connection between genes and settings/signatures.

The log_2_ fold change of each of the “tumor-specific” genes identified in the manual curation for each setting/signature, indicated that AKR1B10 gene is the most upregulated, showing same levels in both “HCC-specific” and “HCV-HCC-specific” signatures. With regards to the three common genes, the CDKN2A and IGF2BP3 genes showed almost same levels of upregulation in all three settings/signatures, whereas the ZNF623 gene showed the highest level in the “HBV-HCC-specific” setting/signature (Fig. 4).

### Validation of the “tumor-specific” genes’ expression by RNA sequencing

**Figure 5:**
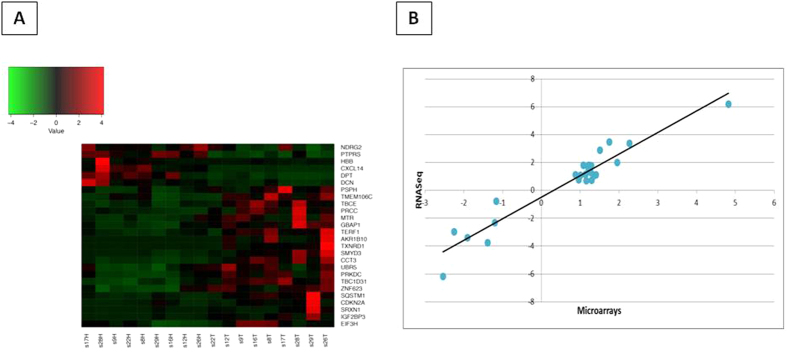
Heat-map of differentially expressed tumor-specific genes by RNA-Seq analysis in HCC and adjacent non tumor tissue samples from nine HCV chronic infected patients (T = tumor sample, H = healthy/non tumor sample) (**A**). Correlation analysis of fold-change transcriptional modulation observed in the microarray and RNA-Seq analyses (**B**).

In order to validate the biological relevance of gene expression data of “tumor-specific” signature resulting from the microarray analyses, nine totally unrelated HCC patients were selected. Both the primary tumor and the paired non-tumor liver specimens from the same patients have been studied using RNA-Sequencing. Considering the most prevalent risk factor in our geographical area, all samples were HCV-associated (Suppl. Table 7). Differential expression analysis carried out on average expression values from HCC vs non-tumor samples confirmed the upregulation (FDR < 0.05) of 20/31 genes belonging to the “HCV-HCC-specific” signature, identified applying our two-step bioinformatics strategy (Fig. 5A). Interestingly, as we used both liver tumor and non-tumor specimens from the same patient in RNA-Seq analysis we could also verify that all differentially expressed genes were significantly up-regulated in all the patients, contributing to strengthen our findings. Moreover, we also found that 6/7 genes down-regulated in the “HCV-HCC-specific” signature were also confirmed by RNA-Seq data, further confirming the specificity and the relevance of our computational approach (Fig. 5A). Fold changes for each of the genes observed in both analyses were highly comparable with an extremely strong correlation (R^2^ = 0.9215) (Fig. 5B).

### Protein expression of “tumor-specific” genes

The level of protein expression for the identified and validated “tumor-specific” genes was evaluated taking advantage of the large immunohistochemistry data on tumor as well as normal tissues available at The Human Protein Atlas (http://www.proteinatlas.org/).

All the proteins encoded by the tumor-specific genes validated by RNA-Seq analysis were found highly expressed in liver cancer tissues (11 different samples in duplicate), although with a high variability. Indeed, considering all the proteins evaluated, the positivity ranged from 30% to 100% (Suppl. Table 8).

However, the majority of the proteins were more or less broadly expressed also in normal tissues (20 different tissues types in duplicate).

**Figure 6:**
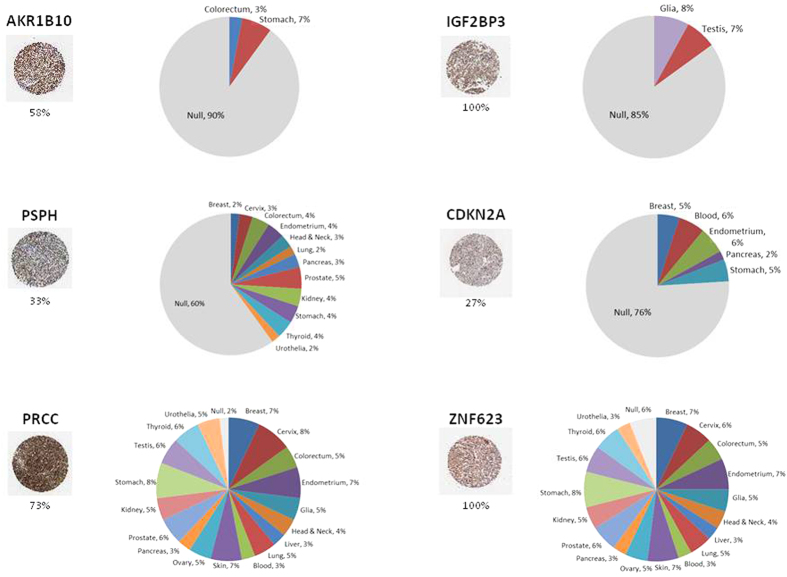
Immunohistochemistry data from The Human Protein Atlas (http://www.proteinatlas.org/). For each of the proteins shown, the IHC image represents an example of expression in HCC tissues and the percentage of expression in all HCC samples is reported. The pie chart represents the percentage of protein expression by IHC in 20 normal tissues.

As examples, the AKR1B10 protein was expressed in 58% of the HCC samples and only in 2/20 normal tissues. On the other extreme, the PRCC protein was expressed in 73% of the HCC samples and in all normal tissues. Considering the three upregulated genes present in both “HBV- and HCV-HCC-specific” signatures, IGF2BP3, and zinc finger protein 623 (ZNF623) were expressed in 100% while Tumor suppressor ARF protein was expressed in 27% of the HCC samples. However, the expression of IGF2BP3 appeared to be highly specific to HCC, given that it was expressed only in 2/20 normal tissues. On the contrary, the ZNF623 protein was expressed in all normal tissues. Tumor suppressor ARF protein showed an intermediate pattern, being expressed in 5/20 normal tissues (Fig. 6).

### Epitope prediction

**Figure 7:**
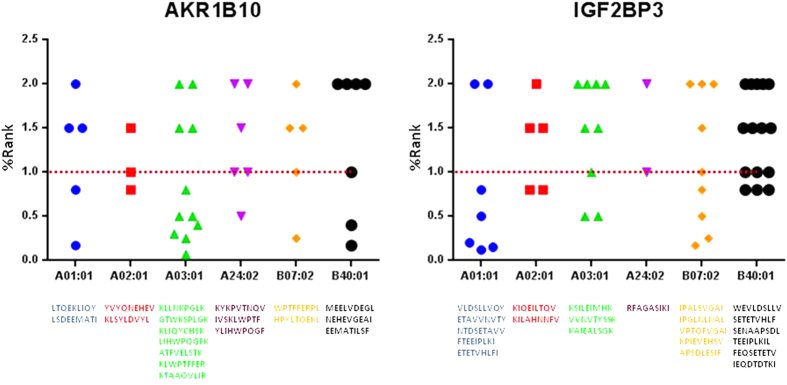
Graphic plotting of predicted epitopes with highest ranking (<2%) associated with indicated HLA class-I alleles. For each allele, each dot represents a predicted epitope. Sequences correspond to epitopes below the dotted line (<1% ranking).

**Figure 8:**
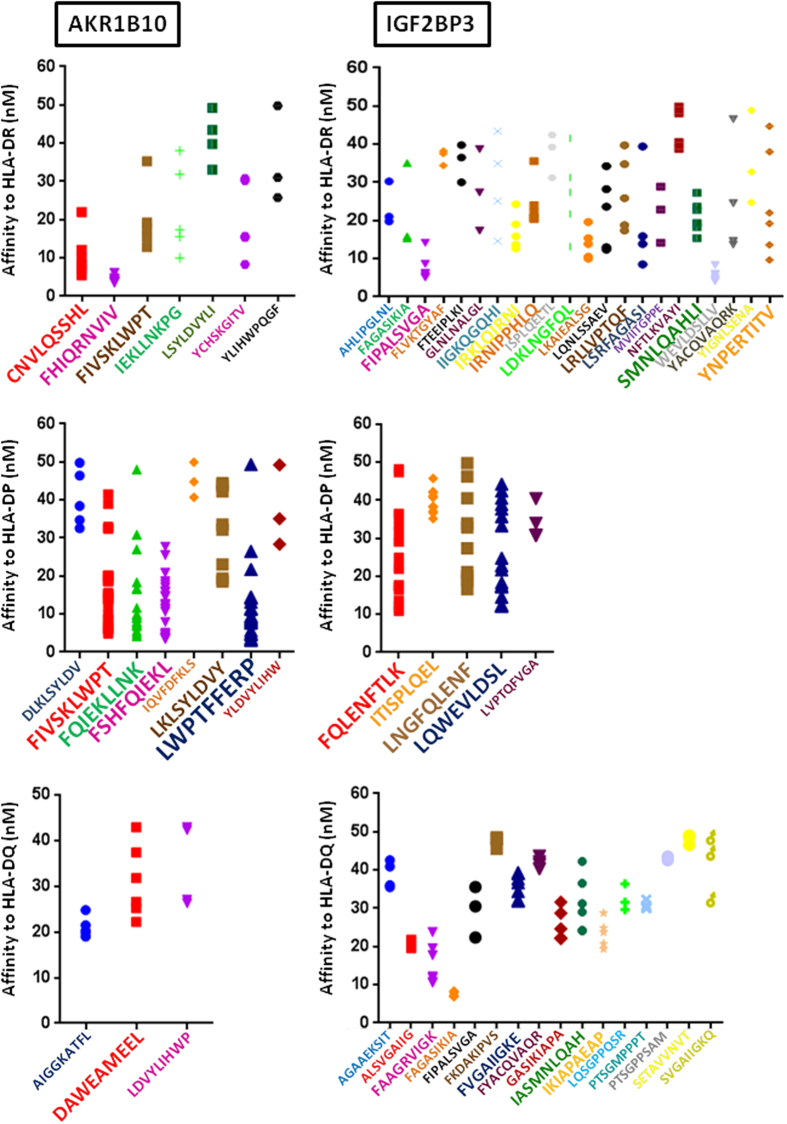
Graphic plotting of predicted epitopes with highest affinity (<50 nM) to the indicated HLA class-II alleles. Each dot represents a predicted epitope covering the corresponding core sequence. Core sequences with less than 3 predicted epitopes have been removed from the analysis. Size of the characters for each core sequence is correlated to the number of predicted epitopes.

In order to predict the possible targeting of highly HCC-specific proteins by immunotherapy strategies, epitope prediction analysis was performed for AKR1B10 and IGF2BP3 proteins. In particular, protein sequences were analyzed for epitope discovery using the prediction tools NetTepi, for HLA class I epitopes, and NetMHCII, for HLA class II epitopes (http://www.cbs.dtu.dk/services/). The *in silico* analysis was performed to predict immunogenic peptides associated with HLA-class I and II alleles covering approximately 90% of the population. A different number of peptides with high rank was predicted for each HLA class I and II alleles. Considering the HLA class I, the average of peptides per alleles was 6.2 (3–11, A2*01 – A3*01) for AKR1B10; and 7.5 (2–15, A24*02 – B40*01) for IGF2BP3. Among those with highest ranking (i.e., <2.0%) for HLA class I alleles, peptides with a more stringent ranking <1.0% are shown (Fig. 7). Considering the HLA class II, more than 360 epitopes have been predicted for AKR1B10 and more than 650 for IGF2BP3. They have been grouped according to core sequences binding to the HLA molecules. A broad difference in number of predicted epitopes for each core sequence was observed. In particular, peptides covering a much broader number of core sequences were predicted for the IGF2BP3 protein. Moreover, for both proteins, a higher number of epitopes per each core sequence was predicted for the HLA-DP alleles (Fig. 8).

## Discussion

A meta-analysis on large datasets of microarray-based transcriptional profiles of HBV- and HCV-associated HCC with non-tumor liver controls and non-tumor tissue types was performed in the present study. Gene expression analysis was carried out in two steps. An initial class comparison of HCC vs. normal liver was performed, and the modulated gene set was used for subsequent comparison with 41 non-tumor tissue types, including liver (Fig. 1). According to such a novel strategy, it was possible to identify “liver-specific”, “tumor-associated” and “tumor-specific” signatures.

In particular, differentially expressed genes identified comparing HCC vs. normal liver were used for subsequent analysis of HCC vs. normal control tissues. Within the 643 upregulated genes, it was possible to identify a “liver-specific” and a “HCC-associated” signature (Fig. 1A). Similar results were obtained when the analysis was conducted with HCC samples stratified for their viral etiology (i.e., HBV and HCV) (Fig. 1B).

Analysis of the “liver-specific” signature confirmed the identification of genes whose functions significantly converged upon liver metabolic pathways and processes (Suppl. Table 2).

IPA analysis of the “tumor-associated” genes identified several molecular pathways common or unique to HBV- and HCV-associated lesions.

In particular, top scoring pathways in HCC were the Estrogen-mediated S-phase Entry Cyclins and Mitotic Roles of Polo-Like Kinase (Suppl. Figure 1). Top scoring pathways in HBV-associated HCC were the Estrogen-mediated S-phase Entry as well as the Cyclins and Cell Cycle Regulation (Suppl. Figure 2).

Interestingly, the top scoring pathway in HCV-associated HCC was the Interferon Signaling. Additional activated pathways were the p53 Signaling and the Estrogen-mediated S-phase Entry (Suppl. Figure 3).

In addition, in all analyses, the pathway of Cell Cycle: G2/M DNA Damage Checkpoint Regulation was characterized by a negative z-score (Suppl. Figures 1–3).

A correlation analysis with toxic functions was, also, performed. IPA showed a significant association with development of hepatotoxicity and liver disorders, such as Liver Hyperplasia/Hyperproliferation (p-value = 4.82E-03) and Hepatocellular Carcinoma (p-value = 9.44E-04) in all settings (i.e., HCC, HBV-HCC and HCV-HCC lesions vs. normal tissues).

Furthermore, correlation between differentially expressed genes and diseases and molecular functions identified a significant association with Cellular Growth and Proliferation, as well as Infectious Diseases.

Finally, analysis of the upstream transcriptional regulators predicted specific activation of several factors involved in cell growth, proliferation and survival.

In particular, our analysis of HCC samples predicted activation of the following factors: E2F1, ERBB2, CCND1, RABL6, HGF, MYC, VEGF, IRF1, TNF, STAT1, JUN, TRAF2, AKT, WNT1, CTNNB1. Interestingly, our results showed a predicted activation for STAT1 which was further confirmed by an exp. Log_2_ ratio of 2.488 (Suppl. Table 3A). Such analysis, also, identified several tumor suppressor genes (i.e., CDKN2A, RB1, let-7, RB, KDM5B, BNIP3L, RBL2, NUPR1, STK11) with negative z-scores^15^ (Suppl. Table 3B).

Interestingly, only in HBV-related HCC the tumor suppressor TP53 and the 26 S Proteasome complex were predicted as inhibited (Suppl. Table 4B).

On the contrary, in HCV-related HCC several members of the IRF family of transcription factors as well as TLRs were predicted as activated (Suppl. Table 5A).

A predicted inhibited state was observed for several miRs. In particular, our results predicted specific inhibition of miR-16, miR-146 and the let-7 family of miRs in HCC. miR-26, miR-124 and miR-155 were inhibited in HBV-associated HCC. miR-24, miR-29, miR-124 and miR-291 were inhibited in HCV-associated HCC. Indeed, a global downregulation of several miRs, during cancer formation, has been reported, and expression of miRs with specific tumor suppressor activities is lost in many tumors^16^,^17^.

Several studies reported a downregulation of the tumor suppressor let-7 family of miRs in HCC^18^. Similarly, miR-26 expression is affected in HCC patients^19^. Also, epigenetic silencing of miR-124 in HCC has been reported. Expression of miR-124 in HCC inhibits cell growth, with direct downregulation of possible targets, including CDK6 and SMYD3^20^. Downregulation of miR-29 in HCC contributes to SETDB1 oncogene up-expression by relieving its post-transcriptional regulation^21^.

Manual curation of the “tumor-associated” genes was performed to identify the “tumor-specific” signatures. Indeed, a subset of 52 genes within the “HCC-associated” signature was selected, representing the “HCC-specific” signature (Fig. 3A). Similarly, “HBV-HCC-specific” as well as “HCV-HCC-specific” signatures were identified (Fig. 3B,C). Interestingly, three genes (i.e., CDKN2A, IGF2BP3 and ZNF623) were common to all three signatures, representing potential markers for HCC regardless the viral etiology.

The altered expression of genes that are included in the “HCC-specific” signature was confirmed by RNA-Seq analysis on nine totally unrelated HCV-associated HCC samples and their non-tumor counterpart. Twenty out of 31 “HCV-HCC-specific” genes selected in our meta-analysis were significantly up-regulated also in RNA-Seq data, and other were just below the threshold of significance used to compute differential expression (FDR < 0.05). Since these findings have been replicated in a completely independent cohort of patients and using a different-and more sensitive-technological approach (i.e., RNA-Seq) we are more confident of the suitability of our two-step bioinformatics strategy. Most importantly, RNA-Seq also validated the upregulation of the three genes identified in both “HBV- and HCV-HCC-specific” signatures (CDKN2A, IGF2BP3 and ZNF623) (Fig. 5A). All of the proteins encoded by the validated tumor-specific genes, including the three common genes, are highly expressed in liver cancer tissues (http://www.proteinatlas.org/) with a variability ranging from 30% to 100%, further confirming the biological relevance of the identified genes. However, most of them were expressed also in a range of normal tissues, confirming the poor correlation between RNA and protein expression profiles^22^,^23^. Only two of them (namely, AKR1B10 present in the “HCV-HCC specific” signature; IGF2BP3 common to both “HBV- and HCV-HCC-specific” signatures) showed a strictly HCC-specific protein expression pattern with no expression in 18/20 normal tissues (Fig. 6). In particular, AKR1B10 is found expressed only in normal tissues of the gastrointestinal (GI) tract, IGF2BP3 is found expressed only in normal glia and testis tissues.

AKR1B10 (Aldo-Keto Reductase Family 1, Member B10) is a member of the aldo/keto reductase (AKR) superfamily whose over-expression has been described in different tumors, including human HCC^24^. More recently, its over-expression has been strongly associated with early stages of HCV-associated hepatocarcinogenesis^25^as well as differentiated HCC^26^. According to such results, it has been proposed as potential target for HCC therapy^27^.

IGF2BP3 (Insulin-Like Growth Factor 2 mRNA-Binding Protein 3) contains several RNA binding domains involved in RNA synthesis and metabolism. It is an oncofetal antigen and as such it may represent a promising target for vaccination^28^. IGF2BP3 expression in HCC is strongly associated with advanced tumor stage, representing a predictor of poor prognosis among HCC patients^29^,^30^.

For both of them, an epitope prediction algorithm identified potential relevant epitopes associated to both HLA-class I and II alleles.

### Conclusions and Future Perspectives

Our analysis strategy represents a proof of concept for the identification of “tumor-specific” genes potentially useful as diagnostic/prognostic markers and/or immunotherapeutic targets. Indeed, “HCC-specific” genes have been identified and validated by RNA-sequencing and immunohistochemistry data, showing a significant correlation between RNA and protein expression. Only two of them (namely, AKR1B10 present in the “HCV-HCC-specific” signature; IGF2BP3 common to both “HBV- and HCV-HCC-specific” signatures) showed a strictly HCC-specific protein expression pattern. The present study is the first demonstration that such genes/proteins relevant in the HCC development and progression are, indeed, highly specific to HCC not being expressed in most of normal tissues. This makes both of them highly potential target for HCC immunotherapy, the AKR1B10 for HBV as well as HCV-associated HCC, the IGF2BP3 for the HBV-associated HCC.

Interestingly, some of the predicted epitopes have been purified from HLA molecules on HCC cells, within the discovery activities of the EU FP7-funded HEPAVAC project (www.hepavac.eu). This conclusively validates the efficacy and specificity of the bioinformatics strategy implemented in the present study. Both class I and II predicted epitopes are undergoing immunological validation.

## Materials and Methods

### Datasets

Gene expression datasets were collected from the Gene Expression Omnibus (GEO) database (available at http://www.ncbi.nlm.nih.gov/geo/). A total of 151 samples from four different datasets (i.e., GSE6764, GSE17548, GSE19665, GSE3526) were selected and integrated to create a unique dataset of interest, including 55 HCC samples (HBV and HCV-related), 14 normal liver and 82 normal samples from 41 different tissue types (Suppl. Table 9).

Samples were selected based on disease condition (i.e., HCC, liver non-tumor controls, non-tumor tissue types), array platform (i.e., Affymetrix HG U133 Plus 2.0) and organized according to etiology (i.e., HBV and HCV).

### Analysis strategy

The identification of the tumor gene signature was based on the workflow schematically represented in Fig. 1.

In the first step, datasets of microarray-based transcriptional profiles of HCC and normal liver were compared. Differentially expressed genes derived from such comparisons were subsequently used for comparison with transcriptional profiles of different normal tissue types.

Finally, based on clustering analysis of differentially expressed genes, “tumor-associated” and “liver-specific” gene subsets were defined. In order to define the “tumor-associated” profile, only genes upregulated in HCC samples with a log_2_ fold change >1 and down-regulated with same expression profiling in the average of normal tissue types were selected. Whereas, to define the “liver-specific” profile only the genes upregulated in both HCC and normal liver samples with a log_2_ fold change >1 were selected.

The “tumor-specific” genes were selected by manual curation of the “tumor-associated” genes. Indeed, “tumor-specific” genes were selected as upregulated in >80% of HCC samples with a log_2_ fold change >1, and downregulated or upregulated in <10% of all normal tissue types with a log_2_ fold change <−1.

### Statistical analysis

Gene expression data were analyzed using BRB-ArrayTools (http://brb.nci.nih.gov/BRB-ArrayTools/) for statistical computing and visualization. Input data were pre-processed with quantile normalization, summarization and adjustment of the background, in order to minimize the variability between samples and to obtain a single intensity value for each probe set. A log_2_ transformation was applied to the data before normalization.

Comparison analyses were subsequently performed and the results were filtered according to fold change and p-value. Differentially expressed genes were selected using a fold change >2 and a p-value associated with a T-test <0.001 in 80% of all the experiments. Gene function was assigned based on Gene Ontology (http://www.geneontology.org/).

### Ingenuity Pathway Analysis

To investigate biological significance of differentially expressed genes, a functional analysis was performed using the Ingenuity Pathway Analysis system (IPA, http://www.ingenuity.com/). For each comparison the most significant canonical pathways were classified according to the lower p-value, and where possible a z-score was applied.

### Enrolled patients and liver samples

Nine HCV chronic infected HCC patients undergoing liver resection were enrolled for the present study (Suppl. Table 7). All human specimens were obtained and processed at the National Cancer Institute in Naples.

The protocol was approved by the National Cancer Institute’s Ethical Committee. Signed informed consent was obtained from all subjects who agreed to provide tissues for the study. All experiments were performed in accordance with relevant guidelines and regulations.

Two paired liver biopsies from each patient with histologically-confirmed HCC and adjacent non-tumor liver tissue were obtained at the time of surgery and stored in RNA-stabilizing agent (RNAlater, Qiagen) for RNA sequencing.

### RNA-Seq: library preparation, sequencing and data analysis

Total RNA was extracted from HCC biopsies using Trizol standard procedure. RNA integrity was assessed using digital gel electrophoresis (Experion, Bio-Rad) and RNA was quantified using spectrophotometer and fluorometer (NanoDrop and Qubit, Life Technologies). Stranded paired-end libraries were prepared using TruSeq Stranded mRNA Sample Preparation Kit (Illumina) and sequenced on Illumina HiSeq2000 platform (100 × 2 bp fragments) as recently described^31^. The computational workflow used to analyze RNA-Seq data has been recently employed^32^. Briefly, reads’ quality was assessed using FastQC (http://www.bioinformatics.babraham.ac.uk/projects/fastqc/). TopHat version 2.0.10^33^ was used to map reads against the reference human genome (hg19 release) and the GENCODE v19 transcripts’ annotation (downloaded from Table Browser of UCSC; http://genome.ucsc.edu) using default parameters. Uniquely mapped reads were used for further quantification analyses. Coverage files were produced using BEDTools and visual inspection of these files on UCSC Genome Browsers was used to assess the overall quality of the experiment and to inspect gene-specific features. Reads’ count was performed using the *FeatureCount* option of RNA-SeqGUI^34^; filtering and normalization were carried out using *Proportional Test* and *FullQuantile* options. Differentially expressed genes were identified using the complex design of DESeq2 implemented in RNA-SeqGUI. The heat-map of differentially expressed genes has been created in R (heatmap.2 function).

### Protein expression data

Protein expression in HCC tissues as well as in 20 normal tissues (for a total of >80 samples) was confirmed according to immunohistochemistry data publicly available at http://www.proteinatlas.org/.

### Epitope prediction

Epitope prediction was performed on “tumor-specific” genes using prediction tools available at http://www.cbs.dtu.dk/services/. The NetTepi 1.0 Server was used to predict HLA class I epitopes. The method integrates three prediction types, peptide-MHC binding affinity, peptide-MHC stability and T-cell propensity^35^,^36^. A wide range of HLA alleles were evaluated, including HLA-A1, HLA-A2, HLA-A3, HLA-A24, HLA-B7 and HLA-B44, covering approximately 90% of the population. Epitopes are predicted based on the combination of the three parameters included in the analyses and ranked on prediction values for 200.000 random natural peptides. The NetMHCII 2.2 Server was used to predict HLA class II epitopes using artificial neural networks. Predictions are obtained for 14 human HLA-DR alleles covering the 9 HLA-DR supertypes, six HLA-DQ and six HLA-DP class II alleles. Prediction values are given in nM IC50 values. Strong binding peptides are those with a <50nM value.

## Additional Information

**How to cite this article**: Petrizzo, A. *et al*. Identification and Validation of HCC-specific Gene Transcriptional Signature for Tumor Antigen Discovery. *Sci. Rep.* **6**, 29258; doi: 10.1038/srep29258 (2016).

## Supplementary Material

Supplementary Information
