# Supplementary material for: Identification and Validation of HCC-specific Gene Transcriptional Signature for Tumor Antigen Discovery
Source: Sci Rep. 2016 Jul 8;6:29258. doi: 10.1038/srep29258 (PMC4937235; doi:10.1038/srep29258)
Supplement: Supplementary Information [file srep29258-s1.docx]

**Identification and Validation of HCC-specific Gene Transcriptional Signature for Tumor Antigen Discovery.**

**Annacarmen Petrizzo^1^^, Francesca Pia Caruso^2,3^^, Maria Tagliamonte^1^, Maria Lina Tornesello^1^, Michele Ceccarelli^2,3^, Valerio Costa^4^, Marianna Aprile^4^, Roberta Esposito^4^, Gennaro Ciliberto^5^, Franco M. Buonaguro^1^, Luigi Buonaguro^1*^**

**Supplementary Table 1.** List of “liver-specific” genes common to all three HCC settings.

| **Symbol** | **Name** | **Accession** | **Defined Genelist** |
| --- | --- | --- | --- |
| ACADSB | acyl-CoA dehydrogenase, short/branched chain | NM_001609 | Fatty acid metabolism, Metabolic pathways, Valine, leucine and isoleucine degradation |
| ACSM5 | acyl-CoA synthetase medium-chain family member 5 | AI733019 | Butanoate metabolism, Metabolic pathways |
| ALDH8A1 | aldehyde dehydrogenase 8 family, member A1 | NM_022568 |  |
| ANGPTL3 | angiopoietin-like 3 | AV659209 |  |
| APOA5 | apolipoprotein A-V | AF202890 | PPAR signaling pathway |
| APOF | apolipoprotein F | NM_001638 |  |
| C1R | complement component 1, r subcomponent | AL573058 | Classical Complement Pathway, Complement Pathway, Complement and coagulation cascades, Phagosome, Staphylococcus aureus infection, Systemic lupus erythematosus |
| C3P1 | complement component 3 precursor pseudogene | AV700829 |  |
| C4orf19 | chromosome 4 open reading frame 19 | AI935586 |  |
| C8A | complement component 8, alpha polypeptide | NM_000562 | Alternative Complement Pathway, Classical Complement Pathway, Complement Pathway, Lectin Induced Complement Pathway, Amoebiasis, Complement and coagulation cascades, Prion diseases, Systemic lupus erythematosus |
| C8B | complement component 8, beta polypeptide | NM_000066 | Amoebiasis, Complement and coagulation cascades, Prion diseases, Systemic lupus erythematosus |
| C9 | complement component 9 | K02766 | Alternative Complement Pathway, Classical Complement Pathway, Complement Pathway, Lectin Induced Complement Pathway, Amoebiasis, Complement and coagulation cascades, Prion diseases, Systemic lupus erythematosus |
| CIDEB | cell death-inducing DFFA-like effector b | NM_014430 |  |
| CYP2C18 | cytochrome P450, family 2, subfamily C, polypeptide 18 | NM_000772 | Arachidonic acid metabolism, Drug metabolism - cytochrome P450, Linoleic acid metabolism, Metabolic pathways, Metabolism of xenobiotics by cytochrome P450, Retinol metabolism |
| CYP2C9 | cytochrome P450, family 2, subfamily C, polypeptide 9 | M21940 | Nuclear Receptors in Lipid Metabolism and Toxicity, Arachidonic acid metabolism, Drug metabolism - cytochrome P450, Linoleic acid metabolism, Metabolic pathways, Metabolism of xenobiotics by cytochrome P450, Retinol metabolism |
| CYP4F2 | cytochrome P450, family 4, subfamily F, polypeptide 2 | D26480 | Arachidonic acid metabolism, Metabolic pathways |
| CYP4V2 | cytochrome P450, family 4, subfamily V, polypeptide 2 | AU146978 |  |
| DGAT2 | diacylglycerol O-acyltransferase 2 | AW469523 | Fat digestion and absorption, Glycerolipid metabolism, Metabolic pathways |
| F11 | coagulation factor XI | NM_000128 | Intrinsic Prothrombin Activation Pathway, Platelet Amyloid Precursor Protein Pathway, Complement and coagulation cascades |
| GBA3 | glucosidase, beta, acid 3 | AW235567 | Cyanoamino acid metabolism, Starch and sucrose metabolism |
| GHR | growth hormone receptor | NM_000163 | AKT Signaling Pathway, Growth Hormone Signaling Pathway, Regulation of eIF4e and p70 S6 Kinase, The IGF-1 Receptor and Longevity, Trefoil Factors Initiate Mucosal Healing, Cytokine-cytokine receptor interaction, Jak-STAT signaling pathway, Neuroactive ligand-receptor interaction |
| GNE | glucosamine (UDP-N-acetyl)-2-epimerase/N-acetylmannosamine kinase | NM_005476 | Amino sugar and nucleotide sugar metabolism, Metabolic pathways |
| GNMT | glycine N-methyltransferase | AF101477 | Glycine, serine and threonine metabolism |
| HGFAC | HGF activator | NM_001528 |  |
| HPX | hemopexin | BC005395 |  |
| KLKB1 | kallikrein B, plasma (Fletcher factor) 1 | NM_000892 | Intrinsic Prothrombin Activation Pathway, Complement and coagulation cascades |
| LINC01146 | long intergenic non-protein coding RNA 1146 | BC029479 |  |
| LOC101928505 | uncharacterized LOC101928505 | BC020897 |  |
| LRG1 | leucine-rich alpha-2-glycoprotein 1 | AA622495 |  |
| MASP2 | mannan-binding lectin serine peptidase 2 | NM_006610 | Complement Pathway, Lectin Induced Complement Pathway, Complement and coagulation cascades, Staphylococcus aureus infection |
| PGLYRP2 | peptidoglycan recognition protein 2 | BE672390 |  |
| PLGLB2 | plasminogen-like B2 | AV688060 |  |
| PON1 | paraoxonase 1 | U53784 | Metabolic pathways |
| PON3 | paraoxonase 3 | L48516 | Metabolic pathways |
| PPP1R3B | protein phosphatase 1, regulatory subunit 3B | W60806 | Insulin signaling pathway |
| RDH16 | retinol dehydrogenase 16 (all-trans) | AF086735 | Metabolic pathways, Retinol metabolism |
| SHMT1 | serine hydroxymethyltransferase 1 (soluble) | L23928 | Cyanoamino acid metabolism, Glycine, serine and threonine metabolism, Metabolic pathways, One carbon pool by folate |
| SLC17A2 | solute carrier family 17, member 2 | NM_005835 |  |
| SLC22A1 | solute carrier family 22 (organic cation transporter), member 1 | NM_003057 | Bile secretion |
| SLC22A7 | solute carrier family 22 (organic anion transporter), member 7 | BC017963 | Bile secretion |
| SLC41A2 | solute carrier family 41 (magnesium transporter), member 2 | BG169689 |  |
| SLC7A2 | solute carrier family 7 (cationic amino acid transporter, y+ system), member 2 | AA876372 |  |
| SLCO1B3 | solute carrier organic anion transporter family, member 1B3 | NM_019844 | Bile secretion |
| SPRYD4 | SPRY domain containing 4 | AI570493 |  |
| STEAP3 | STEAP family member 3, metalloreductase | AF262322 | p53 signaling pathway |
| TTR | transthyretin | AF162690 |  |
| UPB1 | ureidopropionase, beta | AI770035 | beta-Alanine metabolism, Drug metabolism - other enzymes, Metabolic pathways, Pantothenate and CoA biosynthesis, Pyrimidine metabolism |
| XDH | xanthine dehydrogenase | BG260086 | Free Radical Induced Apoptosis, Caffeine metabolism, Drug metabolism - other enzymes, Metabolic pathways, Peroxisome, Purine metabolism |

**Supplementary Table 2.** Gene ontology enrichment analysis of the “liver-specific” genes common to all three HCC settings.

| **GO Biological Process** | **Fold Enrichment** | **P-value** |
| --- | --- | --- |
| complement activation, alternative pathway (GO:0006957) | > 5 | 2.64E-02 |
| complement activation, classical pathway (GO:0006958) | > 5 | 3.34E-04 |
| humoral immune response mediated by circulating immunoglobulin (GO:0002455) | > 5 | 6.35E-04 |
| complement activation (GO:0006956) | > 5 | 1.22E-03 |
| protein activation cascade (GO:0072376) | > 5 | 3.53E-06 |
| regulation of humoral immune response (GO:0002920) | > 5 | 2.80E-02 |
| immunoglobulin mediated immune response (GO:0016064) | > 5 | 7.91E-03 |
| B cell mediated immunity (GO:0019724) | > 5 | 8.89E-03 |
| fatty acid metabolic process (GO:0006631) | > 5 | 1.89E-03 |
| monocarboxylic acid metabolic process (GO:0032787) | > 5 | 4.72E-06 |
| carboxylic acid metabolic process (GO:0019752) | > 5 | 8.10E-09 |
| organic hydroxy compound metabolic process (GO:1901615) | > 5 | 7.34E-03 |
| oxoacid metabolic process (GO:0043436) | > 5 | 6.10E-08 |
| organic acid metabolic process (GO:0006082) | > 5 | 8.13E-08 |
| cellular lipid metabolic process (GO:0044255) | > 5 | 2.18E-05 |
| lipid metabolic process (GO:0006629) | > 5 | 6.44E-07 |
| oxidation-reduction process (GO:0055114) | > 5 | 1.08E-02 |

The second column shows the Fold Enrichment of the genes observed in the uploaded list over the expected. If it is greater than 1, it indicates that the category is overrepresented in the experiment.

**Supplementary Table 3A.** Activated upstream regulators identified in the HCC setting analysis.

| **Upstream Regulator** | **z-score** | **p-value of overlap** | **Target Molecules in Dataset** |
| --- | --- | --- | --- |
| Akt | 2.401 | 1.38E-02 | CXCL10,CXCR4,FOS,IL32,LCN2,MAP2,PCNA,RRM2,SPP1 |
| CCND1 | 3.034 | 2.54E-21 | ASPM,ATAD2,AURKA,CASC5,CCNA2,CCNE2,CDKN2A,CDKN2B,CDKN2C,CENPF,CENPK,CEP55,COL5A2,CPNE3,DEPDC1,DONSON,DTL,FAM83D,GAS2L3,KIAA0101,KIF11,KIF20A,KIF4A,MCM4,MELK,MORC4,MTFR2,MYBL1,PCNA,RACGAP1,RRM2,SPP1,TPX2,TYMS,UHRF1 |
| CTNNB1 | 2.173 | 1.69E-02 | BAMBI,CCNA2,CCNE2,CDKN2A,CDKN2B,COL4A1,ECM1,FOS,GLUL,GNAO1,IRS1,ITGA6,NDRG2,PCDH9,PCNA,PHLDA2,RASSF4,SPP1,SQSTM1 |
| E2F1 | 4.093 | 7.47E-24 | ATAD2,AURKA,BUB3,CCNA2,CCNB1,CCNE2,CDC20,CDK1,CDKN2A,CDKN2C,CSE1L,DBF4,DLEU2,E2F3,ECT2,EGR1,EZH2,FOS,GINS1,GMNN,HELLS,HIST1H2AC,IRS1,KIAA0101,MAD2L1,MCM4,MCM5,MCM6,MSH2,NDC80,NUSAP1,PCNA,PDK1,PRKAA2,PRKDC,RACGAP1,RFC3,RFC4,RRM2,SMC4,TAP2,TOP2A,TP53BP2,TXNRD1,TYMS,UHRF1 |
| ERBB2 | 2.773 | 1.81E-21 | ACSL4,ASPM,AURKA,BAG2,BIRC3,BUB1,BUB1B,CADM1,CCL20,CCNA2,CCNB1,CCNE2,CDC20,CDK1,CDKN2A,CDKN2B,CDKN2C,CDKN3,CENPF,CKS2,COL4A1,COL5A2,CXCL10,DERL1,DPT,EGR1,EPSTI1,FAM134B,FOS,GINS1,ITGA6,LAMP2,LCN2,MYBL1,NCAPG,NDC80,NEDD9,NEK2,PCNA,PHLDA2,PRC1,RAD51AP1,RFC4,RRM2,SMAD2,SQLE,SRPX,TAP2,TOP2A,TYMS,ZFP36L2,ZWINT |
| HGF | 4.199 | 3.90E-19 | ARPC5,AURKA,BAG2,BIRC3,BUB1,BUB1B,CCNE2,CDC20,CDK1,CDKN2A,CDKN2B,CDKN2C,CDKN3,CENPF,COL4A1,CSE1L,CXCR4,DBF4,EGR1,FOS,FOSB,GMNN,HELLS,HMMR,ITGA6,ITGB3BP,KIF11,LCN2,LYVE1,MAD2L1,MCM5,MELK,MID1,NCOA2,NDC80,NEK2,PCNA,PHLDA2,PRC1,PRKDC,SPP1,TPX2,TTK |
| IRF1 | 2.138 | 1.11E-03 | CCNB1,CXCL10,E2F3,GBP2,MAP4K4,NSMCE2,PCNA,STAT1,TAP2 |
| JUN | 2.134 | 9.88E-03 | BIRC3,CCNA2,CDC20,CDK1,CDKN2A,CXCL10,EHD4,FOS,FTH1,HNRNPU,LAMP2,MSH2,SPP1,STAT1 |
| MYC | 3.151 | 8.38E-15 | ACSL4,BOP1,BUB1,BUB1B,CCNA2,CCNB1,CCNE2,CCT3,CDC20,CDK1,CDKN2A,CDKN2B,CKS2,COL4A1,COL5A2,CPD,CXCL10,DLEU2,E2F3,ECM1,EGR1,EZH2,FOS,FTH1,GBP2,GCLM,GLUL,GNPAT,GPC3,HNRNPU,IRS1,ITGA6,LAMP2,LYZ,MAD2L1,MCM5,MCM6,MSH2,MTFR2,NBN,NDRG2,PCNA,PDK1,RARRES1,RRM2,RRM2B,SPP1,TLE1,TP53I3,TSPAN7,TXNRD1,TYMS,UBE2S,XPO1,ZIC2 |
| RABL6 | 4.472 | 2.15E-20 | BUB1,BUB1B,CCNA2,CCNB1,CCNE2,CENPF,EZH2,HMMR,MAD2L1,MCM5,MELK,NCAPG,NDC80,NEK2,PBK,PRC1,RFC3,TOP2A,TPX2,TTK |
| STAT1 | 2.024 | 5.36E-03 | APOBEC3B,CXCL10,EGR1,FOS,GBP2,IFI27,LCN2,RNF213,SMAD2,STAT1,TP53BP2 |
| TNF | 2.354 | 5.23E-03 | AKR1B10,APOBEC3B,BAMBI,BIRC3,BUB1B,CCL20,CDKN2A,CDKN2C,CXCL10,CXCR4,DCN,EGR1,FOS,FOSB,FTH1,GBP2,GCLM,HSPA4,IER2,IFI27,IL32,IRS1,ITGA6,KIF20A,KRT23,LCN2,LIFR,LYVE1,MAP4K4,NCOA2,NEDD9,PTPN12,ROBO1,RRM2,RRM2B,SPP1,SQLE,SQSTM1,STAT1,TP53I3,TXNRD1 |
| TRAF2 | 2.216 | 1.06E-02 | AURKA,BIRC3,CCNB1,CDK1,CDKN2A |
| Vegf | 4.264 | 8.99E-14 | AURKA,BAG2,BUB1,BUB1B,CCNE2,CDC20,CDK1,CDKN2A,CDKN2C,CDKN3,CENPF,CSE1L,CXCL10,CXCR4,DBF4,EGR1,FOSB,GMNN,HELLS,HMMR,ITGA6,ITGB3BP,KIF11,LCN2,LYVE1,MAD2L1,MCM5,MELK,MID1,NCOA2,NDC80,NEK2,PHLDA2,PRC1,TPX2,TTK |
| WNT1 | 2.219 | 1.68E-02 | CCNB1,CDKN2A,EGR1,FOS,SPP1,TLE1 |

**Supplementary Table 3B.** Inhibited upstream regulators identified in the HCC setting analysis.

| **Upstream Regulator** | **z-score** | **p-value of overlap** | **Target Molecules in Dataset** |
| --- | --- | --- | --- |
| BNIP3L | -3.148 | 2.10E-08 | AURKA,CCNA2,CCNE2,CDKN3,CENPF,CKAP2,KIF11,NUF2,RRM2,TOP2A |
| CDKN2A | -3.511 | 1.67E-18 | ATAD2,BUB1B,CCNA2,CDK1,CDKN2A,CDKN2B,CDKN2C,CENPK,CXCL10,CXCL14,DCK,DCTN4,DONSON,E2F3,EGR1,EHD3,EZH2,FOS,IGF2BP3,MAD2L1,MCM4,MCM5,MCM8,MELK,PCNA,RAD51AP1,RFC3,RFC4,RMI1,RRM2,RRM2B,TMPO |
| KDM5B | -3.614 | 9.15E-10 | AURKA,BUB1B,BUB3,CCNB1,CDK1,DLGAP5,ECT2,EGR1,HMMR,NDC80,NEDD9,PBK,PIR,SPTSSA,TOP2A,TTK |
| let-7 | -4.648 | 8.45E-16 | AURKA,BUB1,BUB1B,CCNA2,CCNB1,CCNE2,CDC20,CDK1,DBF4,EZH2,GMNN,MAD2L1,MCM4,MCM5,MCM6,MCM8,NRAS,NUF2,RFC3,RFC4,RRM2,SMAD2 |
| NUPR1 | -2.746 | 1.11E-07 | ABL2,ANP32E,APOBEC3B,ASPM,ATR,AURKA,BUB1,BUB1B,C1orf112,CASC5,CCNA2,CXCR4,FLVCR1,GBP2,GINS1,IGF2BP3,IRF2BP2,KIF11,KIF20A,MTFR2,PDK1,RASAL2,RMI1,SPTSSA,TDRKH,TMPO,TP53BP2 |
| Rb | -3.246 | 1.42E-10 | CCNA2,CCNE2,CDK1,CDKN2A,ECT2,EZH2,GMNN,MAD2L1,MCM5,PCNA,RFC4,TOP2A,TYMS |
| RB1 | -2.704 | 6.74E-18 | ATAD2,CCNA2,CCNB1,CCNE2,CDK1,CDKN2A,CENPK,DCK,DCTN4,DONSON,E2F3,EGR1,EHD3,EZH2,FOS,GMNN,KIAA0101,MCM4,MCM5,MCM6,MCM8,MELK,PCNA,RAD51AP1,RFC3,RFC4,RMI1,RRM2,TMPO,TUBG1,TYMS |
| RBL2 | -2.602 | 7.29E-08 | AURKA,BUB1,BUB1B,CCNA2,CCNB1,CDK1,CDKN2A,KPNA2,NEK2,PCNA,RRM2,TYMS |
| STK11 | -2.219 | 1.25E-01 | CCNA2,ELOVL2,ITGB3BP,NEDD9,TOP2A |

**Supplementary Table 4A.** Activated transcription regulators identified in HBV-HCC setting.

| **Upstream Regulator** | **z-score** | **p-value of overlap** | **Target Molecules in Dataset** |
| --- | --- | --- | --- |
| SOX2 | 2.007 | 1.32E-02 | ATG10,CDKN2A,CXCL14,HHAT,IRF6,PDK1,PEG3,SOX13,TERF1,TXNRD1,ZIC2 |

**Supplementary Table 4B.** Inhibited transcription regulators identified in HBV-HCC setting.

| **Upstream Regulator** | **z-score** | **p-value of overlap** | **Target Molecules in Dataset** |
| --- | --- | --- | --- |
| E2F6 | -2 | 3.13E-03 | GMNN,KIAA0101,MCM5,RAD51AP1,RRM2 |
| TP53 | -2.952 | 1.10E-14 | APBB2,ASPM,ATAD2,ATG10,AURKA,BLZF1,BUB1,BUB1B,CCNB1,CCNE2,CDK1,CDKN2A,CDKN3,CENPF,CEP55,CHMP4C,CKAP2,COL4A1,COL5A2,DBF4,DCK,DLGAP5,ELK4,ESR1,EZH2,FAT1,FIGNL1,FOXP3,GMNN,GNA13,HMMR,KIAA0101,KNTC1,KPNA2,MAD2L1,MCM5,MELK,MIS18BP1,MTDH,MYBL1,NCAPG,NDC80,NDRG2,NEK2,NUSAP1,PBK,PDK1,PEG3,RACGAP1,RAD51AP1,RB1CC1,RFC3,ROBO1,RRM2,RRM2B,SH3BP4,SHC1,SMC4,SMURF1,TMEM43,TOP2A,TP53BP2,TPX2,TTK,XPO1 |

**Supplementary Table 5A.** Activated transcription regulators identified in HCV-HCC setting.

| **Upstream Regulator** | **z-score** | **p-value of overlap** | **Target Molecules in Dataset** |
| --- | --- | --- | --- |
| IFI16 | 2.201 | 2.97E-03 | BAX,CCL20,CDKN2A,CXCL10,ISG15,PCNA |
| IRF3 | 3.24 | 6.14E-07 | APOBEC3B,BIRC3,CXCL10,IFI27,IFI44,IFI6,IFIH1,IFIT3,ISG15,OAS2,OAS3,PARP12,STAT1,TAP1,TMPO |
| IRF5 | 2.559 | 5.71E-08 | BAX,CXCL10,CXCR4,IFI44,IFIH1,IFIT3,ISG15,OAS2,PARP12,STAT1,TMPO |
| IRF7 | 3.91 | 8.98E-08 | CXCL10,IFI44,IFI6,IFIH1,IFIT3,ISG15,MICB,MX1,OAS2,OAS3,PARP12,RTP4,STAT1,TAP1,TAP2,TMPO |
| NFE2L2 | 2.947 | 1.90E-02 | AKR1B10,CCT3,CDKN2C,CXCL10,FTH1,HSP90AB1,HTATIP2,LGALS8,PSMD14,PTGS2,SQSTM1,SRXN1,TXN,TXNRD1 |
| STAT2 | 2.18 | 2.71E-05 | CXCL10,IFI27,IFI6,IFIT3,ISG15,MX1,OAS2 |

**Supplementary Table 5B.** Inhibited transcription regulators identified in HCV-HCC setting.

| **Upstream Regulator** | **z-score** | **p-value of overlap** | **Target Molecules in Dataset** |
| --- | --- | --- | --- |
| CREBBP | -2.213 | 2.64E-03 | CXCL10,CXCR4,EGR1,EGR3,EPSTI1,FOS,FOSB,HBB,ISG15,ITGA6,MSH2,OAS3,PCNA,PTGS2,RTP4,TAP2 |
| HMGA1 | -2.219 | 1.41E-01 | COL4A1,EGR1,FOS,IER2,PTGS2 |
| NANOG | -2 | 6.37E-02 | EGR1,FOS,FOSB,HNRNPH1,SMARCC1 |
| NUPR1 | -3.024 | 1.38E-07 | ABL2,ANP32E,APOBEC3B,ASPM,ATR,AURKA,BUB1,BUB1B,C1orf112,CASC5,CCNA2,CXCR4,E2F8,FLVCR1,GBP2,GINS1,IGF2BP3,IRF2BP2,KIF11,KIF20A,LMNB1,MTFR2,PDK1,RASAL2,RMI1,SPTSSA,TDRKH,TMPO,TP53BP2 |
| SIRT1 | -2.2 | 1.23E-01 | BAX,BIRC3,CDKN2A,CDKN2B,NEDD4L,PER1,PRMT3 |

**Supplementary Table 6.** Networks including “tumor-specific” genes.

| **Tumor-Specific Gene** | **Setting** | **Networks** | **Modulated Genes** | **Modulation** |
| --- | --- | --- | --- | --- |
| **CDKN2A**  **IGF2BP3** | HBV-HCC | Cell Cycle, DNA Replication, Recombination, and Repair, Cellular Response to Therapeutics | ATR, CDK1, CDK12, **CDKN2A,** COL4A1, Cyclin A, cytochrome C, DCK, DLGAP5, DTL, EZH2, FRY, GM-CSF, **IGF2BP3,** MCM5, MCM8, MDK, MED14, mediator, NBPF15, PARP, PI3K (complex), PLOD3, PRKDC, RAD1, RAD51AP1, RFC3, RNApolymerase II, SMYD3, TLE1, WDR26, ZHX1, ZIC2  XIST  RPA |  |
|  | HCV-HCC | Cell Cycle, Post-Translational Modification, Cellular Development | BOP1, CCNA2, CCNE2, CDC73, CDK4/6-Cyclin D1, **CDKN2A,** CDKN2B, CDKN2C, Cyclin A, Cyclin E, DONSON, HIST1H1C, HIST1H2AC, Histone H1, **IGF2BP3,** INK4, MDK, MELK, MTR, PSMD, PSMD10, PTTG1, Smad, UHRF1, WDR26, ZNF217  DACH1, EHD3  cytochrome C, Cyclin D, Cyclin D1/cdk4, MEF2, PI3K (complex), PDGF-AA, NCK |  |
| **ZNF623** | HBV-HCC | Developmental Disorder, Hereditary Disorder, Organismal Functions | EFCAB2, FKTN, GLMP, LINS1, RASEF, SDE2, SLC18B1, TGIF1, TMEM68, TMEM209, TMEM38B, UBAP2L, ZBTB41, **ZNF623**  API5, BAG3, DAG1, DOK4, ELAVL1, GNAZ, LGALS8, MAT2B, MRPS10, MSI1, MUM1, PAIP2, POMGNT1, PRPS2, PTRF, PVRL3, RAB2B, RABL2B, RAPSN, ZBTB3, ZNF426 |  |
|  | HCV-HCC | Antimicrobial Response, Inflammatory Response, Dermatological Diseases and Conditions | APOBEC3B, BLZF1, CKS2, EHD4, IFI6, IFI27, IFI44, IFIH1, IFIT3, Interferon-α Induced, ISG15, ISGF3, MX1, Oas, OAS2, OAS3, RNF213, STAT1, TAP1, TAP2, **ZNF623**  Ifn, IFN alpha/beta, IFN Beta, IFNgamma, IFN type 1, Ifnar, Interferon alpha, IL12 (complex), ERK, IL-2R, MHC Class I (complex), JAK, SOCS, TMSB4 |  |

**Supplementary Table 7.** Enrolled HCV chronic infected HCC patients.

| **Patient Number** | **Age** | **Gender** | **Liver Parameters (U/L)** | | | | **TNM Stage** | **Child-Pugh** | **Grading** |
| --- | --- | --- | --- | --- | --- | --- | --- | --- | --- |
|  |  |  | **AST** | **ALT** | **GGT** | **ALP** |  |  |  |
| 8 | 73 | F | 90 | 85 | 82 | 105 | T2 N0 M0 | A | G2 |
| 9 | 72 | M | 84 | 76 | 43 | 91 | T2 N0 M0 | A | G2 |
| 12 | 57 | F | 55 | 97 | 33 | 110 | T3 N0 M0 | A | G2 |
| 16 | 65 | M | 68 | 110 | 117 | 128 | T1 N0 M0 | B | G2 |
| 17 | 71 | M | 37 | 27 | 31 | 90 | T3 N0 M1 | B | G2 |
| 22 | 70 | M | 33 | 57 | 34 | 82 | T3 N0 M0 | B | G2 |
| 26 | 76 | M | 50 | 70 | 51 | 99 | T2 N0 M0 | A | G2 |
| 28 | 65 | M | 82 | 155 | 65 | 97 | T3 N0 M0 | A | G2 |
| 29 | 71 | M | 57 | 47 | 199 | 302 | T3 N0 M0 | B | G2 |
|  |  |  |  |  |  |  |  |  |  |

**Supplementary Table 8.** Percentage of HCC samples positive for expression of the indicated tumor-specific proteins. Data are derived from the Human Protein Atlas (<http://www.proteinatlas.org/>).

| Tumor-Specific Protein | Ab1 | Ab2 | Ab3 |
| --- | --- | --- | --- |
| ZNF623 | 7/7 |  |  |
| TXNRD1 | 2/6 | 6/6 | 6/6 |
| IGF2BP3 | 7/7 |  |  |
| CCT3 | 6/6 |  |  |
| PRKDC | 7/7 | 6/6 |  |
| SMYD3 | 4/4 |  |  |
| SQSTM1 | 6/8 |  |  |
| UBR5 | 7/7 |  |  |
| PRCC | 1/8 | 3/6 | 6/6 |
| TBC1D31 | 5/8 |  |  |
| AKR1B10 | 7/8 | 6/7 |  |
| PSPH | 5/8 | 1/8 |  |
| EIF3H | 8/8 | 3/8 |  |
| TMEM106C | 1/7 |  |  |
| TBCE | 6/8 | 8/8 | 4/8 |
| CDKN2A | 1/8 | 4/7 | 1/6 |

**Supplementary Table 9.** List of samples included in the study.

| **Experiment Name** | **Tissue Type** | **Viral Etiology** | **Diagnosis** |
| --- | --- | --- | --- |
| GSM155919 | Liver | NA | Normal tissue |
| GSM155926 | Liver | NA | Normal tissue |
| GSM155927 | Liver | NA | Normal tissue |
| GSM155928 | Liver | NA | Normal tissue |
| GSM155932 | Liver | HCV | Very early HCC |
| GSM155933 | Liver | HCV | Very early HCC |
| GSM155934 | Liver | HCV | Very early HCC |
| GSM155935 | Liver | HCV | Very early HCC |
| GSM155936 | Liver | HCV | Very advanced HCC |
| GSM155937 | Liver | HCV | Early HCC |
| GSM155938 | Liver | HCV | Advanced HCC |
| GSM155939 | Liver | HCV | Early HCC |
| GSM155940 | Liver | HCV | Advanced HCC |
| GSM155941 | Liver | HCV | Advanced HCC |
| GSM155942 | Liver | HCV | Advanced HCC |
| GSM155943 | Liver | HCV | Very advanced HCC |
| GSM155944 | Liver | HCV | Very advanced HCC |
| GSM155945 | Liver | HCV | Early HCC |
| GSM155946 | Liver | HCV | Early HCC |
| GSM155947 | Liver | NA | Normal liver tissue |
| GSM155948 | Liver | NA | Normal liver tissue |
| GSM155949 | Liver | HCV | Very advanced HCC |
| GSM155950 | Liver | HCV | Very advanced HCC |
| GSM155953 | Liver | HCV | Very advanced HCC |
| GSM155954 | Liver | HCV | Very advanced HCC |
| GSM155955 | Liver | HCV | Early HCC |
| GSM155956 | Liver | HCV | Very advanced HCC |
| GSM155957 | Liver | HCV | Very advanced HCC |
| GSM155958 | Liver | HCV | Early HCC |
| GSM155959 | Liver | HCV | Early HCC |
| GSM155960 | Liver | HCV | Early HCC |
| GSM155961 | Liver | NA | Normal tissue |
| GSM155962 | Liver | HCV | Very advanced HCC |
| GSM155963 | Liver | HCV | Advanced HCC |
| GSM155964 | Liver | NA | Normal tissue |
| GSM155983 | Liver | HCV | Very early HCC |
| GSM155985 | Liver | HCV | Advanced HCC |
| GSM155986 | Liver | HCV | Early HCC |
| GSM155987 | Liver | HCV | Very early HCC |
| GSM155988 | Liver | NA | Normal tissue |
| GSM155989 | Liver | NA | Normal tissue |
| GSM155990 | Liver | HCV | Advanced HCC |
| GSM155991 | Liver | HCV | Very early HCC |
| GSM155992 | Liver | HCV | Early HCC |
| GSM155993 | Liver | HCV | Very early HCC |
| GSM437458 | Liver | HBV | HCC |
| GSM437459 | Liver | HBV | HCC |
| GSM437461 | Liver | HCV | HCC |
| GSM437464 | Liver | HBV | HCC |
| GSM437467 | Liver | HBV | HCC |
| GSM437468 | Liver | HBV | HCC |
| GSM437474 | Liver | HBV | HCC |
| GSM437489 | Liver | HCV | HCC |
| GSM437491 | Liver | HBV | HCC |
| GSM437493 | Liver | HBV | HCC |
| GSM490988 | Liver | HBV | Moderately differentiated HCC |
| GSM490990 | Liver | HBV | Moderately differentiated HCC |
| GSM490992 | Liver | HCV | Moderately-poorly differentiated HCC |
| GSM490994 | Liver | HCV | Moderately differentiated HCC |
| GSM490996 | Liver | HBV | Poorly differentiated HCC |
| GSM490998 | Liver | HCV | Moderately differentiated HCC |
| GSM491000 | Liver | HCV | Moderately differentiated HCC |
| GSM491002 | Liver | HCV | Moderately differentiated HCC |
| GSM491004 | Liver | HBV | Moderately differentiated HCC |
| GSM491006 | Liver | HBV | Moderately-poorly differentiated HCC |
| GSM80576 | Bone Marrow | NA | Normal tissue |
| GSM80577 | Bone Marrow | NA | Normal tissue |
| GSM80578 | Bronchus | NA | Normal tissue |
| GSM80579 | Bronchus | NA | Normal tissue |
| GSM80580 | Adipose Tissue | NA | Normal tissue |
| GSM80583 | Adipose Tissue | NA | Normal tissue |
| GSM80591 | Amygdala | NA | Normal tissue |
| GSM80592 | Amygdala | NA | Normal tissue |
| GSM80595 | Putamen | NA | Normal tissue |
| GSM80596 | Putamen | NA | Normal tissue |
| GSM80605 | Adrenal Gland Cortex | NA | Normal tissue |
| GSM80606 | Adrenal Gland Cortex | NA | Normal tissue |
| GSM80609 | Coronary Artery | NA | Normal tissue |
| GSM80610 | Coronary Artery | NA | Normal tissue |
| GSM80617 | Cerebellum | NA | Normal tissue |
| GSM80618 | Cerebellum | NA | Normal tissue |
| GSM80625 | Colon Cecum | NA | Normal tissue |
| GSM80632 | Colon Cecum | NA | Normal tissue |
| GSM80633 | Cervix | NA | Normal tissue |
| GSM80634 | Cervix | NA | Normal tissue |
| GSM80641 | Cerebral Cortex | NA | Normal tissue |
| GSM80642 | Cerebral Cortex | NA | Normal tissue |
| GSM80654 | Heart Atrium | NA | Normal tissue |
| GSM80655 | Heart Atrium | NA | Normal tissue |
| GSM80657 | Heart Ventricle | NA | Normal tissue |
| GSM80658 | Heart Ventricle | NA | Normal tissue |
| GSM80673 | Endometrium | NA | Normal tissue |
| GSM80674 | Endometrium | NA | Normal tissue |
| GSM80687 | Kidney Crtex | NA | Normal tissue |
| GSM80688 | Kidney Crtex | NA | Normal tissue |
| GSM80691 | Hypothalamus | NA | Normal tissue |
| GSM80692 | Hypothalamus | NA | Normal tissue |
| GSM80694 | Esophagus | NA | Normal tissue |
| GSM80697 | Esophagus | NA | Normal tissue |
| GSM80699 | Midbrain | NA | Normal tissue |
| GSM80700 | Midbrain | NA | Normal tissue |
| GSM80707 | Lung | NA | Normal tissue |
| GSM80710 | Lung | NA | Normal tissue |
| GSM80713 | Medulla | NA | Normal tissue |
| GSM80714 | Medulla | NA | Normal tissue |
| GSM80717 | Myometium | NA | Normal tissue |
| GSM80718 | Myometium | NA | Normal tissue |
| GSM80725 | Mammary Gland | NA | Normal tissue |
| GSM80726 | Mammary Gland | NA | Normal tissue |
| GSM80728 | Liver | NA | Normal tissue |
| GSM80729 | Liver | NA | Normal tissue |
| GSM80730 | Liver | NA | Normal tissue |
| GSM80732 | Kidney Medulla | NA | Normal tissue |
| GSM80733 | Kidney Medulla | NA | Normal tissue |
| GSM80736 | Lymph Nodes | NA | Normal tissue |
| GSM80737 | Lymph Nodes | NA | Normal tissue |
| GSM80739 | Liver | NA | Normal tissue |
| GSM80748 | Pharyngeal Mucosa | NA | Normal tissue |
| GSM80749 | Pharyngeal Mucosa | NA | Normal tissue |
| GSM80759 | Ovary | NA | Normal tissue |
| GSM80776 | Oral Mucosa | NA | Normal tissue |
| GSM80777 | Oral Mucosa | NA | Normal tissue |
| GSM80780 | Ovary | NA | Normal tissue |
| GSM80786 | Spinal Cord | NA | Normal tissue |
| GSM80787 | Spinal Cord | NA | Normal tissue |
| GSM80790 | Skeletal Muscle | NA | Normal tissue |
| GSM80791 | Skeletal Muscle | NA | Normal tissue |
| GSM80805 | Prostate Gland | NA | Normal tissue |
| GSM80806 | Prostate Gland | NA | Normal tissue |
| GSM80814 | Stomach Pyloric | NA | Normal tissue |
| GSM80815 | Stomach Pyloric | NA | Normal tissue |
| GSM80822 | Salivary Gland | NA | Normal tissue |
| GSM80823 | Salivary Gland | NA | Normal tissue |
| GSM80825 | Spleen | NA | Normal tissue |
| GSM80826 | Spleen | NA | Normal tissue |
| GSM80838 | Thalamus | NA | Normal tissue |
| GSM80839 | Thalamus | NA | Normal tissue |
| GSM80864 | Thyroid Gland | NA | Normal tissue |
| GSM80865 | Thyroid Gland | NA | Normal tissue |
| GSM80868 | Testes | NA | Normal tissue |
| GSM80869 | Testes | NA | Normal tissue |
| GSM80874 | Vagina | NA | Normal tissue |
| GSM80886 | Tonsil | NA | Normal tissue |
| GSM80887 | Trachea | NA | Normal tissue |
| GSM80888 | Trachea | NA | Normal tissue |
| GSM80889 | Tonsil | NA | Normal tissue |
| GSM80897 | Vulva | NA | Normal tissue |
| GSM80898 | Vulva | NA | Normal tissue |
| GSM80903 | Vagina | NA | Normal tissue |
| GSM80911 | Urethra | NA | Normal tissue |
| GSM80912 | Urethra | NA | Normal tissue |
